# Comparison of percutaneous endoscopic discectomy and microendoscopic discectomy in treatment of symptomatic lumbar disc herniation: A protocol of cohort study

**DOI:** 10.1097/MD.0000000000022709

**Published:** 2020-10-16

**Authors:** Yabin Hu, Yong Zheng, Guangfu Chen, Wei Chen

**Affiliations:** a Department of Orthopedics, The Second Hospital of Nanjing, Jiangsu; b Department of Bone injury, Shaanxi Hospital of Traditional Chinese Medicine, Shaanxi; c Department of Anesthesiology, Fuzhou Second Hospital Affiliated to Xiamen University, Fujian; d Department of Spine Surgery, Fuzhou Second Hospital Affiliated to Xiamen University, Fujian, China.

**Keywords:** lumbar disc herniation, microendoscopic discectomy, percutaneous endoscopic discectomy, protocol

## Abstract

**Background:**

Despite some researchers have compared the safety and effectiveness of percutaneous endoscopic discectomy (PED) and microendoscopic discectomy (MD) for the lumbar disc herniation; however, they got conflicting outcomes in several variables. Therefore, our aim was to clarify whether PED produces less surgical trauma and better clinical results than MD.

**Methods:**

A single-center, retrospective cohort trial was conducted for the comparison of the safety and effectiveness between the MD and PED in the patients with lumbar disc herniation who received surgery from May 2016 to July 2018 in our hospital. The inclusion criteria for our investigation included:

1. age of 30 to 60 years;
2. preoperative CT and MRI scans revealed disc herniation;
3. conservative treatment was unsuccessful for at least 6 weeks;
4. patients with no former lumbar surgery history at same level.

The follow-ups were performed 6 weeks, 3, 6, 12 and 24 months after the surgery. Numeric Rating Scale, Short-form 36, and Oswestry Disability Index, as well as complications were evaluated in our study. The software of SPSS Version 22.0 (IBM Corporation, Armonk, NY) was applied to analyze all the statistical data. When *P* is less than .05, the difference is significant in statistics.

**Results:**

This protocol will provide a solid theoretical basis for exploring which technique is better in treatment of lumbar disc herniation.

**Trial registration:**

This protocol was registered in Research Registry (researchregistry6005).

## 1 Introduction

The pain in the leg and back after lumbar disc herniation is the result of compression and an uncomfortable process caused by a herniated disc. It is a kind of familiar disease, and its prevalence rate is approximately 2% to 5% in general population.^[1–4]^ Although most patients with lumbar disc herniation obtained the pain relief through pharmaceutical treatment and physical therapy and other conservative treatment, there are still nearly 40% to 60% of the patients require surgical treatment.^[5,6]^

With the development of minimally invasive surgery, a variety of minimally invasive discectomy has been introduced. Microendoscopic discectomy (MD), a popular surgical technique to treat the lumbar disc herniation, was first proposed via Smith and Foley in 1997, and it has become more and more popular among the spinal surgeons.^[7–10]^ Many researches have demonstrated that it is safe and effective even when applied in the treatment of the recurrent lumbar disc herniation. Nevertheless, in MD, it is inevitable that the laminabony structure and the spine tension band will be destroyed, which may cause low back pain and lumbar instability after operation.^[11–15]^ In the past few years, the percutaneous endoscopic discectomy (PED) has increasingly become a common treatment for the symptomatic lumbar disc herniation. In numerous former reports, it possesses a good therapeutic effect on lumbar disc herniation. This kind of procedure seems to be superior to the microendoscopic discectomy because it can be carried out under the condition of local anaesthesia and it is less invasion. The meta-analysis and systematic review indicated that the clinical results of MD and PED are comparable.^[16–21]^

Despite some researchers have compared the safety and effectiveness of PED and MD for the lumbar disc herniation; however, they got conflicting outcomes in several variables. Therefore, our aim was to clarify whether PED produces less surgical trauma and better clinical results than MD. Postoperative follow-up was conducted to acquire various clinical indicators results and then these results were compared in order to offer basis for the clinical treatment.

## 2 Materials and methods

### 2.1 Population

The inclusion criteria for our investigation included:

1. age of 30 to 60 years;
2. preoperative CT and MRI scans revealed disc herniation;
3. conservative treatment was unsuccessful for at least 6 weeks;
4. patients with no former lumbar surgery history at same level.

The exclusion criteria contained:

1. the patients with serious disc calcification, severe and moderate spinal canal stenosis, lumbar instability as well as posterior vertebral interruption;
2. patients with a former history of the lumbar disc surgery;
3. with poor conditions of local skin or obviously abnormality of laboratory results;
4. the patients did not have complete imaging data or could not complete the follow-up.

### 2.2 Study design

A single-center, retrospective cohort trial was conducted for the comparison of the safety and effectiveness between the MD and PED in the patients with lumbar disc herniation who received surgery from May 2016 to July 2018 in our hospital. This investigation was registered through the research registry (https://www.researchregistry.com/), and its registration no. is researchregistry6005. The clinical research was approved with the clinical research ethics committee of the Fuzhou Second Hospital Affiliated to Xiamen University (FZ100427), all the patients who participated in the research offered the written informed consent.

### 2.3 Techniques

This study was completed by the same operating and nursing team according to the standard medical process. General anesthesia was given for the MD group and local anesthesia for the PED group.

#### 2.3.1 PED technique

The patients in PTED group were kept lateral, and then with the patients legs bent, they were lying on an unaffected side. In accordance with the guidance of C-arm fluoroscopy, from the entrance point, the 18-gauge needle was inserted to lateral hole. Then the 22-gauge needle was inserted into the herniated intervertebral disc through 18-gauge needle, and the contrast agent was injected into the intervertebral disc. Continuous insertion of the dilators was utilized to expand the bony foramen properly. Then identifying and removing the blue degenerated disc material through utilizing endoscopic forceps until nerve root was fully decompressed.

#### 2.3.2 MD technique

The operation in MD group was carried out in the mattress prone position with the incision of 18 mm. The skin was contracted laterally, the sequential dilators and guide wire were placed beside the vertebra and placed under the control of lateral fluoroscopy. A tubular retractor was attached and then secured to the flexible arm. Under the microscope, the unilateral discectomy and lutectomy were conducted. Ultimately, the drainage tubes were placed and then sutured.

### 2.4 Postoperative outcomes

The follow-ups were performed 6 weeks, 3, 6, 12 and 24 months after the surgery. These tests were carried out through 2 physicians in the clinic of authors. In addition to the routine examination, additional information were acquired through applying some parameters, including the pain scale of Numeric Rating Scale, Short-form 36 (SF-36), and the score of Oswestry Disability Index (ODI), as well as complications.

The SF-36 determines 8 indicators: physical pain, role physiology, physical function, social function, vitality, and general health, mental health and role emotional. We chose the SF-36 average bodily pain score and average physical score to perform the analysis. ODI includes 10 items about the severity of leg or back diseases that influence the ability to manage daily living. These 10 components include the daily functions and pain (containing personal hygiene, pain intensity, sitting, walking, lifting, sleeping, standing, and traveling, sexual activity, as well as social activity). Each item will be scored on a six-point scale (0–5); the higher the score, the higher the degree of disability associated with the lower back pain. (Table 1).

**Table 1:**
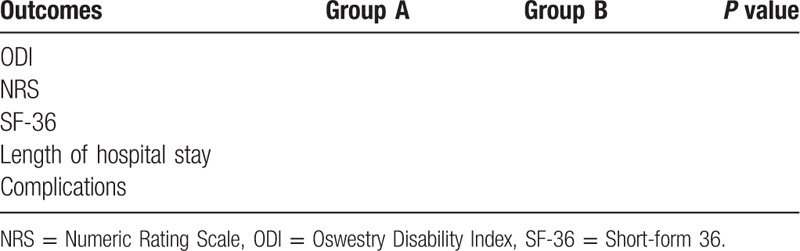
Postoperative outcomes.

### 2.5 Statistical analysis

The software of SPSS Version 22.0 (IBM Corporation, Armonk, NY) was applied to analyze all the statistical data. All the data of determination were indicated as a mean ± SD. We chose *t*-test for comparison among groups and compared the normal distribution data of different points in time within group and analyzed the variance of data through repeated measure. We adopted the χ^2^ test for enumeration data. And then the rank sum detection was also utilized. When *P* is less than .05, the difference is significant in statistics.

## 3 Discussion

Lumbar disc herniation is a frequently occurring and familiar disease. Patients generally appear reduced muscle strength, numbness, low back pain as well as other clinical symptoms, which affect the life quality of patients seriously. Hence, if the conservative treatment fails, it is necessary to choose surgical treatment. Essentially, the surgical treatment is decompression nerve root, specifically the removal of herniated disc nucleus. MD is implemented through applying the posterior transpedicular method, which is easy to operate and conforms to most habits of orthopedics. As for the PED, it is a kind of minimally invasive approach for the treatment of lumbar disc herniation by the lateral method. Hence, we performed this present retrospective cohort trial at the aim of determining the microendoscopic discectomy and percutaneous endoscopic discectomy priority in treating the symptomatic lumbar disc herniation.

## Author contributions

Wei Chen and Yong Zheng conceived, designed, and planed the study. Wei Chen, Yong Zheng, and Guangfu Chen are recruiting the study participants and performing the interventions. Yabin Hu supervised the study. Yabin Hu, Wei Chen and Yong Zheng will interpret and analyze the data. Yabin Hu drafted the manuscript. Guangfu Chen critically revised the manuscript for important intellectual content. All authors have full access to the manuscript and take responsibility for the study design. All authors have approved the manuscript and agree with submission.

**Conceptualization:** Yabin Hu.

**Data curation:** Yabin Hu.

**Formal analysis:** Yabin Hu, Yong Zheng.

**Funding acquisition:** Wei Chen.

**Investigation:** Yong Zheng, Guangfu Chen.

**Methodology:** Guangfu Chen.

**Project administration:** Wei Chen.

**Resources:** Wei Chen.

**Software:** Yong Zheng.

**Supervision:** Wei Chen.

**Validation:** Yong Zheng.

**Visualization:** Guangfu Chen.

**Writing – original draft:** Yabin Hu.

**Writing – review & editing:** Wei Chen.
